# Modulation of Inflammatory and Redox Responses by Saturnia Spa Water and Mud Extract in Human Skin Cell Models

**DOI:** 10.1155/bmri/5993121

**Published:** 2026-10-02

**Authors:** Erica Costantini, Giovanni Pellacani, Chman Shahzadi, Marcella Reale, Shamsa Kanwal, Giuseppe Tafuri, Manela Scaramuzzino, César Viseras, Bruna Sinjari

**Affiliations:** ^1^ Department of Innovative Technologies in Medicine and Dentistry University “G. d′Annunzio” Chieti Italy; ^2^ Dermatology Clinic Department of Clinical Internal Anesthesiological and Cardiovascular Sciences La Sapienza University of Rome Roma Italy uniroma1.it; ^3^ Center of Advanced Studies and Technology (CAST) University of “G. d′Annunzio” Chieti–Pescara Italy; ^4^ University School for Advanced Studies IUSS Pavia Pavia Italy; ^5^ Science and Technology for Sustainable Development Department of Innovative Technologies in Medicine and Dentistry University “G. d′Annunzio” Chieti–Pescara Italy; ^6^ Department of Pharmacy and Pharmaceutical Technology Faculty of Pharmacy University of Granada Granada Spain ugr.es; ^7^ Parco Acqua Santa Siena Italy

## Abstract

**Background:**

Thermal waters and peloid‐derived products have long been used in dermatological and spa treatments, although the cellular mechanisms underlying their biological effects remain incompletely characterized. This study investigated the effects of Saturnia spa water (TW) and an aqueous mud extract (MUD) on inflammatory, redox, and wound‐healing responses in human skin cell models.

**Methods:**

Human dermal fibroblasts (NHDF) and keratinocytes (HaCaT) were exposed to different concentrations of MUD and TW to assess cell viability by MTT assay. Based on the viability results, 5% MUD and TW were selected for subsequent experiments. TNF‐*α*–stimulated cells were used to investigate inflammatory and oxidative responses by evaluating IL‐1*β* and IL‐6 production, inflammatory and cytoprotective gene expression, reactive oxygen species (ROS), superoxide dismutase (SOD) activity, and glutathione (GSH) levels. A scratch assay was performed to evaluate the effects of the treatments on cell migration and wound repair.

**Results:**

MUD and TW showed no marked cytotoxic effects at concentrations up to 5% in either cell model, whereas the undiluted preparations affected cell viability. In TNF‐*α*–stimulated NHDF cells, both MUD and TW significantly reduced IL‐1*β* and IL‐6 gene expression (*p* < 0.001), whereas MUD significantly reduced the secretion of both cytokines. Both treatments also enhanced HO‐1 expression and restored the TNF‐*α*–induced reduction in SOD activity, whereas total ROS and GSH levels were not significantly altered. In HaCaT cells, TNF‐*α* approximately doubled ROS levels (*p* < 0.001), whereas both MUD and TW significantly reduced ROS production (*p* < 0.01). TNF‐*α* also reduced SOD activity by approximately 50% (*p* < 0.001), an effect that was restored by both treatments. GSH levels remained unchanged. In the scratch assay, both extracts significantly promoted wound closure at 6 h in NHDF cells (*p* = 0.002) and in HaCaT cells (MUD *p* = 0.017 and TW *p* = 0.027).

**Conclusions:**

Under the experimental conditions tested, Saturnia water and its aqueous mud extract exerted anti‐inflammatory and redox‐modulating effects in human keratinocytes and dermal fibroblasts, together with a positive effect on in vitro wound closure. These findings support further investigation of these spa‐derived preparations as potential complementary agents for skin repair and inflammatory modulation, although their disease‐specific effects require validation in more physiologically relevant models.

## 1. Introduction

The skin is a complex organ that protects the body from various external insults, including UV radiation, temperature fluctuations, pathogens, irritants, pollution, and lifestyle‐related exposures such as body creams, textiles, and allergens from animals and plants. Skin diseases and chronic wounds are conditions that affects the structure, function, or appearance of the skin. In both conditions, inflammation, infections, or immune system problems can lead to symptoms like redness, itching, and irritation that are highly prevalent, affecting approximately one‐third of the global population [1, 2]. The physiological skin response involves antioxidant defenses, antimicrobial activity, mechanical resistance, permeability regulation, ultraviolet filtration, and inflammation as a protective mechanism against harmful insults [3]. Fibroblasts and keratinocytes contribute to both the physical barrier and the host′s immune defense, playing a critical role in skin homeostasis and the response to noxious stimuli. These cells are major contributors to inflammatory responses in the skin, producing cytokines (e.g., IL‐1, TNF‐*α*, IL‐6, G‐CSF, TGF‐*β*, and IL‐10), chemokines (e.g., IL‐8 and IP‐10), growth factors (e.g., IL‐6, GM‐CSF, and TGF‐*α*), and antimicrobial peptides (e.g., *β*‐defensins, cathelicidin, S100 proteins, and sebum) [4, 5]. They constitutively express the pattern recognition receptors (PRRs), such as toll‐like receptors (TLRs), able to recognize pathogen components, followed by the activation of the transcription factor nuclear factor‐kappa B (NF‐*κ*B) that leads to the activation of inducible nitric oxide synthase (iNOS), cyclooxygenase‐2 (COX2), and production of secondary mediators.

Moreover, keratinocytes interact with immune cells, such as macrophages and neutrophils, and act as antigen‐presenting cells that cooperate with T lymphocytes in antigen‐specific and cell‐mediated responses. Thus, they play a pivotal role in both innate and adaptive immunity [6].

TNF‐*α* is a key cytokine involved in various inflammatory skin diseases. It orchestrates the cellular response to stimuli and injury by regulating inflammatory gene expression and modulating cell motility, leukocyte infiltration, the cell cycle, and skin hyperproliferation [7–10]. TNF‐*α* triggers a series of intracellular events that determine the activation of transcription factors, including NF*κ*B, AP‐1, CCAAT enhancer‐binding protein *β* (C/Ebp *β*), responsible for the induction of genes involved in several biological processes, such as cell growth and death, oncogenesis, and immune, inflammatory, and stress responses [7].

Oxidative stress in the skin—due to excessive ROS production and reduced antioxidant defenses—contributes to proto‐oncogene activation, collagen degradation, lipid peroxidation, increased membrane permeability, and chronic inflammation through ROS persistence. Current biological treatments for chronic inflammatory skin diseases include anti–TNF‐*α* agents, selective interleukin antagonists (e.g., IL‐4, IL‐12/23, and IL‐17), and JAK/STAT inhibitors, which effectively downregulate excessive immune responses but are often associated with high costs [11].

In recent years, there has been renewed interest in natural remedies such as thermal products, medicinal herbs, and aromatic plants. An increasing body of evidence supports their beneficial role in promoting skin health and treating dermatological disorders [11, 12]. As reported by Kaptaner and Aytac [13], the aqueous extract of *Hypericum pseudolaeve* at a noncytotoxic concentration of 62 *μ*g/mL significantly enhances cell migration compared with the control, likely due to its high content of epicatechin and antioxidant phenolic compounds. Similarly, Vijithsingh et al. [14], using the human endothelial cell line EA.hy926 as a model to investigate angiogenesis‐related wound healing, demonstrated that the aqueous extract of *M. cordata* exerts anti‐inflammatory effects through the inhibition of the NF‐*κ*B and MAPK signaling pathways. Fronza et al. [15] showed that *Calendula officinalis* extract influences both cell migration and proliferation in 3T3 murine fibroblasts cultured on Type I collagen matrices. In addition, Hormozi et al. [16] reported that treatment with *C. officinalis* leads to an increased expression of growth factors (TGF‐*β*1 and bFGF) within the first 12 h, followed by a reduction after 24 h. This temporal modulation of growth factor expression may contribute to improved fibroblast function during the wound healing process.

Thermal therapy, a branch of natural medicine, has been used for centuries, especially in regions with mineral‐rich springs. Applications of thermal water and mud (balneotherapy) are commonly employed to alleviate chronic musculoskeletal and rheumatic pain by improving blood circulation and joint mobility. The mineral composition of these products is also reported to have immunomodulatory effects, particularly in immune‐mediated skin disorders [11, 12].

Saturnia spa, located in Tuscany, Italy, has been renowned since Etruscan and Roman times for its therapeutic properties. Its thermal sulfurous water, emerging from a single natural spring at 37.5°C, flows into natural limestone pools.

This study investigates the biological effects of Saturnia spa thermal products, specifically mud aqueous extract (MUD) and Saturnia spa water (TW) on TNF‐*α*–induced inflammation in cultured keratinocytes and fibroblasts, aiming to evaluate their antioxidant, anti‐inflammatory, and wound‐healing properties.

## 2. Materials and Methods

### 2.1. Materials

Mud and thermal water sampling was conducted in the Saturnia spa (Tuscany, Italy) during September 2024. Samples were collected using sterile polyethylene containers and stored in a refrigerated container at 4°C until reaching the laboratories, where the mud and water samples were aliquoted and processed for the various experiments.

### 2.2. Chemical Characterization of Saturnia Spa Water

The cationic composition of thermal water was determined to evaluate mineral content and ionic profile. Samples, collected in clean, acid‐washed polyethylene bottles, were filtered through 0.45‐*μ*m membranes to remove particulate matter and stored at 4°C until analysis. The concentrations of major cations were quantified using ICP‐OES PERKIN‐ELMER OPTIMA 8300 (PerkinElmer, Waltham, Massachusetts, United States), following standard protocols. For each analysis, calibration curves were prepared using certified standard solutions of the respective cations. Measurements were performed in triplicate to ensure accuracy and reproducibility. The results were expressed in mg/L of each cation.

### 2.3. Mineralogical and Chemical Characterization of Mud

The mineralogical and chemical characteristics of mud were determined to verify sample identity, purity, and composition. X‐ray diffraction (XRD) analysis was conducted using a Philips X′Pert diffractometer (Philips, The Netherlands) equipped with an automatic slit (CuK*α* radiation, 4°–70° 2*θ* range, scanning rate 6°/min, 40 kV). Analyses were performed on aggregates oriented (AO), mounts (i.e., oriented aggregates prepared to enhance the identification of phyllosilicate clay minerals). Mineral quantification was based on peak area measurements from diffractograms, calibrated using complementary chemical compositional data and processed with the XPOWER program.

The infrared spectra of the samples were recorded in the mid‐IR range (400–4000 cm^−1^) using a Perkin Elmer 783 Fourier transform infrared spectrometer. Data processing was performed with Origin 6 software.

Major element composition was determined by x‐ray fluorescence (XRF) using a Bruker S4 Pioneer spectrometer (Bruker, Massachusetts, United States) with a Rh X‐ray tube (60 kV, 150 mA).

### 2.4. Thermal Analysis of Mud (MUD)

Thermogravimetric (TGA) and derivative thermogravimetric (DTG) analyses measuring, respectively, the change in mass of a sample as a function of temperature and showing the rate of weight loss were applied to determine the thermal stability, content, composition, and decomposition of materials.

The mud analysis was conducted using a Mettler Toledo TGA/DSC1 system (Greifensee, Switzerland) equipped with an FRS5 sensor and a microbalance (precision 0.1 *μ*g) in an air atmosphere. Samples were heated from 25°C to 950°C at a constant rate of 10°C/min.

### 2.5. Preparation of Saturnia Spa Water and Mud Aqueous Extract for Cell‐Based In Vitro Evaluation

Saturnia spa water (TW) was filtered using a Millipore 0.22‐*μ*m vacuum‐driven filter (Millipore, Billerica, Massachusetts) to remove microorganisms and sand. The sample filtrate was used for experiments (TW). Mud extract preparation followed the Spilioti et al. [17] procedure. In brief, 2 g of mud was suspended in 20 mL of distilled water for 48 h on an orbital shaker, then centrifuged at 4000 rpm for 20 min, and the supernatant was filtered using a Millipore 0.22‐*μ*m vacuum‐driven filter (Millipore, Billerica, Massachusetts) and used as mud aqueous extract (MUD). All Saturnia spa samples were sterilized by UV irradiation and used for the following experiments at 37°C.

### 2.6. Cell Viability

The normal human dermal fibroblasts (NHDFs) and human keratinocytes (HaCaT) cells were cultured in Dulbecco′s modified Eagle medium (DMEM) supplemented with 10% fetal bovine serum (FBS), 100 U/mL penicillin and 100 *μ*g/mL streptomycin in a humidified incubator at 37°C in a 5% CO_2_. At confluency, cells were trypsinized and plated into 96‐well plates (Corning, New York, United States) at a density 5 × 10^3^ cells/well. Cells were incubated in serum‐free culture medium for 18 h to synchronize the culture. After the overnight incubation, cells were exposed to MUD and TW (from 100% v/v to 0.5% v/v) for 24 h. As negative control, untreated cells were plated only in presence of growth medium, whereas as positive control DMSO (100% v/v) was added to the cells. After time runs out 10 *μ*L of the 3‐(4,5‐Dimethyl‐2‐thiazolyl)‐2,5‐diphenyl‐2H‐tetrazolium bromide solution (MTT Assay, Merck KGaA, Darmstadt, Germany) was added and cells were incubated at 37°C for 2 h. The culture supernatant was removed and replaced with 100 *μ*L of DMSO to dissolve the formazan crystals. Absorbance was then measured at 560 nm using a microplate spectrophotometer (Glomax, Promega, IT), and data were normalized to the untreated control group, considered as 100% of viability.

### 2.7. Cell Migration Assay

To study the effect of MUD and TW on cell migration, NHDF and HaCaT were cultured in a sixwell plate (0.6 × 10^6^/well) and after reaching the confluence, were TNF‐*α*–treated (R&D Systems, Wiesbaden, Germany) for 6 h, to induce cell inflammation. At the end of TNF‐*α* incubation, a scratch was performed using a sterile tip of 10 *μ*L (Ø = 0.1 mm) following an imaging template to reproduce the wound in the same well position. After scratching cells were washed with PBS (Merck KGaA, Darmstadt, Germany) to remove cell debris, imaged by a contrast phase inverted microscope (Leica DMi1, Wetzlar, Germany) and then incubated at 37°C and 5% CO_2_, in fresh medium in presence of MUD or TW at 5%.

After 6 and 24 h, 10x objective pictures were collected using a microscope guide mask to capture the same area in each well. The percentage of the cell‐free area was calculated by referring to images at T0 of scratch for each sample. The wound healing was compared between TNF*α*‐treated samples and TW or MUD‐treated samples by measuring the wound width, using the digital images analyzing software ImageJ (National Institutes of Health, Bethesda, Maryland, United States).

### 2.8. Cell Culture, Treatments, and Gene Expression Analysis

To study the effect of MUD and TW on the inflammatory gene expression, NHDF and HaCaT were seeded in sixwell plates (0.6 × 10^6^ cells/well) and, upon reaching confluence, were treated with TNF‐*α* for 6 h and at the end of the incubation period, cultures were supplemented with MUD or TW (5%).

The cells were lysed with TRIZOL reagent (Invitrogen Technologies, Carlsbad, California, United States), and the total RNA was isolated following the classical acid guanidinium thiocyanate‐phenol‐chloroform extraction method. RNA content and quality were measured by Nanodrop Spectrophotometer (Thermo Fisher Scientific, United States), and subsequent cDNA was synthesized using the QuantiTect Reverse Transcription Kit (Qiagen, United States). To assess the relative mRNA levels, SYBR Green reaction (Promega, Milan, Italy) was performed using the CFX96 System (Bio‐Rad, United States) for the amplification of 18s, IL‐6, IL‐1*β*, iNOS, COX‐2, and HO‐1 primers (Supporting Information 1: Table of primer sequences). The data were analyzed using the delta cycle threshold method and 2^−*Δ* *Δ* *C* *T*^ method, and the expression levels of each gene were normalized to 18s (Ribosomal Protein S18) and determined as fold change of the TNF‐*α*–treated group. All values are reported as means ± standard deviation (SD).

### 2.9. Cell Culture, Treatments, and Enzyme‐Linked Immunosorbent Assays (ELISA)

To measure the concentration of IL‐1*β* and IL‐6 the ELISA assay was performed. After incubation in the presence of TNF‐*α* (6 h) and Saturnia spa extracts (MUD and TW) for 24 h, supernatant was collected and immediately stored until assay execution.

A human IL‐1*β* and IL‐6 ELISA kit (R&D Systems, United States) was applied according to the manufacturer′s instructions. The optical density at 450 nm was measured using a microplate reader GloMax‐Multi Detection System (Promega Corporation, United States). Cytokine concentration was calculated using a standard reference curve. The intra‐assay and interassay reproducibility was > 90%. The specificity and the sensitivity of the cytokines were defined according to the manufacturer.

### 2.10. Cell Culture, Treatments, and ROS Determination

To determine the ROS levels in HaCaT and NHDF the 2 ^′^, 7 ^′^‐Dichlorofluorescin diacetate (DCFH‐DA) solution (Merck KGaA, Darmstadt, Germany) was used. Cells were cultured in a specific complete medium with a density of 15,000 cells/well, and cultured at 37°C in a humidified atmosphere of 5% CO_2_. When the cell confluence level reached 80% and after the medium change, TNF *α* (50 ng/mL) was added for 6 h and then supplemented with Saturnia spa extracts, until 24 h.

At the end of the incubation, 5M DCFH‐DA was added for 30 min. The dichlorofluorescein (DCF) resulting compound, produced by the reaction of dichlorodihydrofluorescein with the ROS, possibly present in cultured and treated cells, can be detected using a multiplate reader.

DCF was detected at a wavelength of 540 nm by microplate reader GloMax‐Multi Detection System (Promega Corporation, United States). DCFH‐DA–unloaded cells were examined in parallel and subtracted from DCFH‐DA–loaded cell values.

### 2.11. Cell Culture, Treatments, and Superoxide Dismutase (SOD) Determination

SOD activity was determined using a commercial assay kit (Catalog Number 19160, Merck KGaA, Darmstadt, Germany), according to the manufacturer′s instructions. This assay is based on the ability of SOD to inhibit the reduction of a tetrazolium salt (WST‐1) by superoxide anions generated enzymatically via the xanthine/xanthine oxidase system. Superoxide radicals are generated enzymatically in the reaction system, and the extent of inhibition is proportional to the SOD activity present in the sample. The SOD was measured in cell culture supernatants after cell treatments, as previously described in presence of TNF*α* and MUD extract and TW. Absorbance was measured spectrophotometrically at 440 nm, and SOD activity represented the percentage of the inhibition of water‐soluble tetrazolium salt (WST‐1) through the measure of the decrease in color development.

### 2.12. Cell Culture, Treatments, and GSH Determination

Total glutathione (GSH + GSSG) levels were determined using a commercial assay kit (Merck KGaA, Darmstadt, Germany; Cat. No. CS0260), following the manufacturer′s instructions. Briefly, NHDF and HaCaT cells (2.5 × 10^6^ cells/mL) were treated with TNF‐*α* alone or in combination with MUD and TW, harvested and deproteinized using 5% sulfosalicylic acid, and centrifuged to remove precipitated proteins. The assay is based on a kinetic enzymatic recycling method in which glutathione reduces 5,5 ^′^‐dithiobis‐(2‐nitrobenzoic acid) (DTNB) to form the yellow chromophore 5‐thio‐2‐nitrobenzoic acid (TNB). The oxidized glutathione (GSSG) generated in the reaction is subsequently reduced back to GSH by glutathione reductase in the presence of NADPH, allowing continuous cycling.

The rate of TNB formation, directly proportional to the total glutathione concentration in the sample, was monitored spectrophotometrically at 412 nm using a microplate reader GloMax‐Multi Detection System (Promega Corporation, United States). Glutathione levels were calculated using a standard curve and expressed as nmol/mg protein.

### 2.13. Statistical Analysis

Statistical analyses were conducted using both parametric and nonparametric approaches, as appropriate. Differences among experimental groups were assessed by one‐way analysis of variance (ANOVA) followed by the Kruskal–Wallis multiple comparisons test. An independent‐samples Student′s *t*‐test was used to compare the means between the two groups. Differences were considered significant at (*p* < 0.05).

## 3. Results

### 3.1. Chemical Characterization of Saturnia Spa Water and Mud of Saturnia

The physicochemical properties of Saturnia spa water analyzed in this study are summarized in Figure 1a. The therapeutic significance of this water is largely attributed to its high sulfur content. Trace elements such as boron (~4.5 mg L^−1^), lithium (~0.18 mg L^−1^), and strontium (~8–9 mg L^−1^) were detected, whereas elevated sulfate concentrations reflect subsurface sulfide oxidation or gypsum dissolution. Bicarbonate and calcium derive from carbonate dissolution and CO_2_ interaction, and the presence of dissolved H_2_S and free CO_2_ indicates a reducing, CO_2_‐rich reservoir environment. Hydrochemically, the water belongs to the Ca–Mg–SO_4_–HCO_3_ facies, consistent with prolonged water–rock interaction in a deep carbonate–evaporitic aquifer capped by low‐permeability clays. Active degassing and travertine depositions at the spring indicate supersaturation with respect to calcite. The water exhibits high mineralization, with elevated concentrations of calcium (605.6 mg L^−1^), sodium (94.77 mg L^−1^), magnesium (59.75 mg L^−1^), and sulfur (> 650 mg L^−1^), classifying it as a calcium–sodium sulfate thermal water. These features are characteristic of deep‐circulating thermal systems and align with hydrogeochemical models of major European thermal springs [18]. The trace element profile, including lithium, strontium, fluoride, boron, and silicon (12.89 mg L^−1^), reflects interaction with carbonate and silicate rocks, whereas iron was below detection limits, consistent with reducing conditions. Overall, this mineral‐rich water supports its established therapeutic applications; however, its high salinity and scaling potential may limit its suitability for drinking or agricultural use.

**Figure 1:**
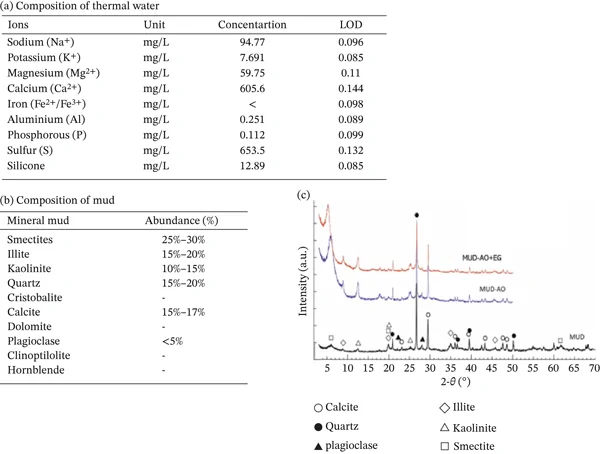
Saturnia spa products composition. (a) Spectrometer evaluation of Saturnia spa water. (b) X‐ray diffraction (XRD) patterns of mud. (c) Diffractogram of mud. AO, aggregates oriented; EG, ethylene glycol treatment.

The XRD pattern of raw mud Figure 1b,c shows a heterogeneous mineral composition dominated by quartz (SiO_2_) and calcite (CaCO_3_), with characteristic reflections at ~26°–27° and ~29°–30° 2*θ*, respectively. Lower intensity peaks attributable to plagioclase, illite, kaolinite, and smectite were also observed, indicating the presence of a clay fraction with potential cation‐exchange and swelling properties. Following AO preparation (i.e., oriented clay aggregates mounting for XRD analysis) mud aggregates oriented (mud‐AO), a slight decrease in the intensity of clay mineral reflections was detected, whereas quartz and calcite peaks remained largely unchanged. This suggests partial modification of the clay lattice, likely associated with reduced crystallinity and preferential orientation effects. In the mud‐aggregates oriented‐ethylene glycol (mud‐AO + EG) sample, where ethylene glycol (EG) solvation treatment was applied to assess expandable clay phases, particularly smectite, clay mineral peaks exhibited further broadening and intensity reduction, indicating a pronounced loss of crystallinity and increased amorphous character. In contrast, quartz reflections persisted, confirming the structural stability of the crystalline framework. Overall, the XRD results indicate that mud comprises both crystalline and clay minerals, and that AO preparation and EG solvation selectively affect the structural ordering of the clay without affecting the dominant quartz and calcite phases. These structural modifications may enhance surface reactivity and functional properties relevant to cosmetic and balneological applications.

The FTIR spectrum of mud provides valuable insights into its mineralogical and chemical composition (Supporting Information 2: FTIR spectrum of mud). The broad band observed between 3600–3400 cm^−1^ is attributed to the stretching vibrations of “‐OH” groups, typically associated with both structural hydroxyls in clay minerals and adsorbed water molecules. The peak near 1630–1650 cm^−1^ is indicative of bending vibrations from water molecules, further confirming the hydrated nature of the sample. This feature is common in smectite and montmorillonite clay, which have high water content due to their expansive interlayer spaces. In the region around 1100–1000 cm^−1^, strong absorption bands are observed, characteristic of Si–O stretching vibrations. Such bands are typical markers of silicate frameworks in clays like montmorillonite, illite, or kaolinite. A distinct doublet or shoulders in this region may point to a mixture of layered silicates with some structural variety or minor quartz admixture, which is also present in natural muds. The absorption seen near 800–500 cm^−1^ may correspond to Al–O bending vibrations, confirming the presence of aluminosilicate minerals predominant in muds and natural clays. These spectral features together point to a composition dominated by hydrated aluminosilicate minerals with minimal organic or carbonate impurities. Such a mineralogical profile is ideal for pharmaceutical use, conferring high cation exchange capacity, adsorption potential, and stability to Saturnia mud, as demanded for advanced drug delivery and topical formulations [19, 20].

The XRF analysis indicates that mud is predominantly siliceous and aluminous, with high SiO_2_ (51.21%) and Al_2_O_3_ (14.83%) contents, consistent with a clay‐ and silicate‐rich composition (Table 1). CaO (10.41%), along with moderate MgO (2.73%) and Fe_2_O_3_ (6.78%), suggests contributions from carbonate phases, mafic silicates, and iron‐bearing minerals. Minor alkali oxides (K_2_O and Na_2_O) and low TiO_2_, MnO, and P_2_O_5_ reflect accessory mineral components. The trace element profile shows enrichment in Sr (1081.67 ppm) and Ba (433.67 ppm), likely associated with carbonate or hydrothermal inputs. Transition metals (V, Cr, Ni, Cu, and Co) indicate contributions from mafic or oxide phases, whereas Zr and Y suggest the presence of refractory accessory minerals. Additional trace elements (Rb, Nb, Pb, and Th) provide a distinct geochemical signature, whereas Rh was below the detection limit. Overall, mud samples exhibit a siliceous–aluminous composition with moderate Ca, Mg, and Fe contents and enrichment in selected trace metals, consistent with a volcanic–sedimentary origin. These characteristics support its suitability for cosmetic and therapeutic applications, where mineral composition and trace element content are relevant.

**Table 1:** Major and trace element composition of Saturnia mud determined by x‐ray fluorescence (XRF).

Elements	Unit	Concentration
V	Ppm	166.00
Cr	Ppm	155.33
Co	Ppm	33.67
Ni	Ppm	86.33
Cu	Ppm	77.33
Zn	Ppm	240.00
Ga	Ppm	28.67
Rb	Ppm	277.67
Sr	Ppm	1081.67
Y	Ppm	75.67
Zr	Ppm	459.00
Nb	Ppm	51.67
Rh	Ppm	—
Ba	Ppm	433.67
Pb	Ppm	50.00
Th	Ppm	33.67
Na_2_O	%	0.49
MgO	%	2.73
Al_2_O_3_	%	14.83
SiO_2_	%	51.21
K_2_O	%	2.39
CaO	%	10.41
TiO_2_	%	0.82
MnO	%	0.19
Fe_2_O_3_	%	6.78

*Note:* Major oxides are reported in weight percent (%), trace elements in parts per million (Ppm).

### 3.2. Thermal Analysis of Mud

TGA and DTG curves of mud exhibit a typical multistep thermal decomposition pattern of clay‐rich natural muds (Figure 2). An initial mass loss up to ~150°C (~46.7%) corresponds to the removal of physically adsorbed and interlayer water. Between 150°C and 400°C, only minor mass loss (~0.6%) occurs, likely from low‐temperature volatiles or minor organics. A broader loss between 400°C and 700°C (~2.1%) reflects dehydroxylation of structural hydroxyl groups in clay minerals. Finally, a small mass‐loss step from 700°C to 900°C is attributed to carbonate decomposition, primarily calcite. Beyond this, the curves stabilize, indicating completion of major thermal events. Overall, mud contains significant bound and structural water, minor organics, dehydroxylating clay phases, and carbonate fractions.

**Figure 2:**
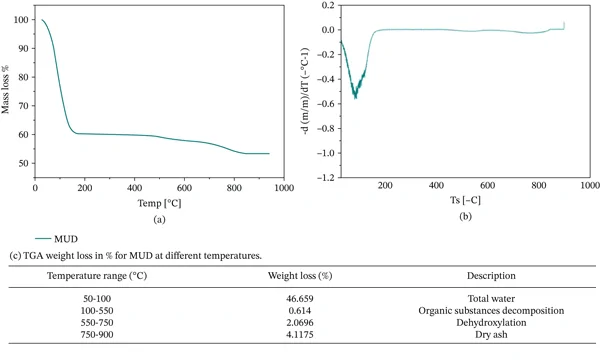
Thermal analysis of mud. (a) TGA reported as mass loss (%) as a function of temperature. (b) DTG curve expressed as the first derivative of the normalized mass loss with respect to temperature [−d(m/m₀)/dT] and plotted as a function of temperature (°C). (c) TGA weight loss in % for MUD at different temperatures.

### 3.3. Effects of MUD and TW Extracts on Cell Viability

Under standard in vitro culture conditions, NHDF and HaCaT cell lines exhibited normal growth characteristics. To assess the cytotoxicity of MUD and TW extracts, NHDF and HaCaT cells were treated for 24 h with concentrations ranging from 0.5% to 100%, and cell viability was evaluated by the MTT assay. As reported in Figure 3a,b, in NHDF cells, no statistically significant changes in viability were observed across the tested concentrations, with the exception of the undiluted 100% MUD and TW preparations, which produced a marked reduction in cell viability compared with untreated cells. A similar trend was observed in HaCaT cells, in which no significant changes in viability were detected following treatment with MUD or TW at the tested concentrations, whereas the highest concentration resulted in reduced viability (Figure 3c,d).

**Figure 3:**
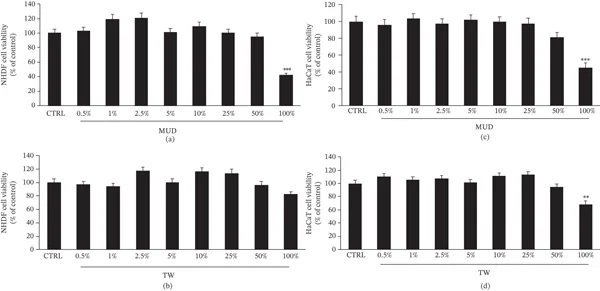
Cell viability. (a) NHDF cells were treated with MUD, (b) NHDF cells were treated with TW, (c) HaCaT cells were treated with MUD, and (d) HaCaT cells were treated with TW. For MUD and TW were tested from 100% v/v to 0.5% v/v for 24 h. MTT assay data were reported as the mean ± SD of three independent experiments, expressed as a percentage of viable cells to untreated cells (CTRL = 100*%*of viability). Statistical differences were evaluated between treatments using Student *t*‐test.

Overall, these findings indicate that the effects of MUD and TW on cell viability were limited at concentrations below 100%. Based on the absence of significant cytotoxicity and the consistency of the response in both cell models, 5% MUD and 5% TW were selected for all subsequent experiments.

### 3.4. Effects of MUD and TW Extracts on Inflammation and Oxidative Stress in TNF‐*α*–Stimulated NHDF and HaCaT Cells

The evaluation of the effect of the treatments, MUD and TW, was conducted on the TNF*α*‐induced inflammation model. As shown in Figures 4 and 5, the graphs show the effect in relation to changes in mRNA levels (fold change Panel a) in relation to TNF*α* and the evaluation of protein levels and enzymatic/oxidative activity (Panels b and c).

**Figure 4:**
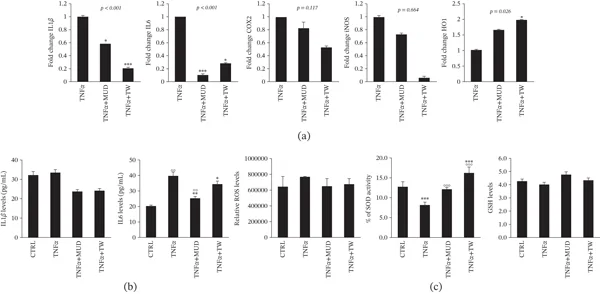
NHDF TNF‐*α*–stimulated response to MUD and TW extracts. (a) Graphs of mRNA expression of NHDF; (b) IL‐1*β* and IL6 levels in NHDF; and (c) levels of ROS, SOD activity, and GSH in NHDF. Data are expressed as mean ± SEM of two independent experiments in duplicates. One‐way ANOVA and Kruskal–Wallis multiple comparisons test, for unmatched comparisons were performed for the statistical analysis (∗*p* < 0.05; ∗∗*p* < 0.01; and ∗∗∗*p* < 0.001). For ELISA results, One‐way ANOVA and Kruskal–Wallis multiple comparisons test were performed for the statistical analysis (∗*p* < 0.05; ∗∗*p* < 0.01 vs. TNF*α*‐treated samples; °*p* < 0.05; °°*p* < 0.01 vs. untreated samples). For oxidative stress evaluation, the Student *t*‐test was performed for statistical significance ∗*p* < 0.05; ∗∗*p* < 0.01; ∗∗∗*p* < 0.001 vs. untreated samples; °*p* < 0.05; °°*p* < 0.01; °°°*p* < 0.001 vs. TNF*α*‐treated samples.

**Figure 5:**
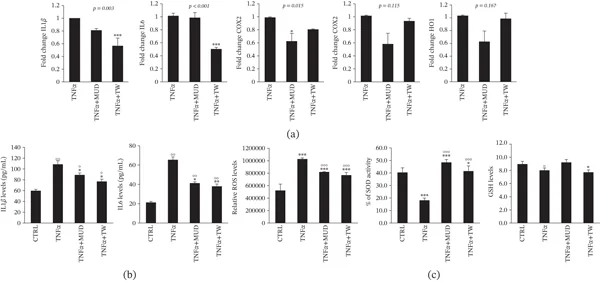
HaCaT TNF‐*α*–stimulated response to MUD and TW extracts. (a) Graphs of mRNA expression of HaCaT; (b) IL‐1*β* and IL6 levels in HaCaT; and (c) levels of ROS, SOD activity, and GSH in HaCaT. Data are expressed as mean ± SEM of two independent experiments in duplicates. One‐way ANOVA and Kruskal–Wallis multiple comparisons test, for unmatched comparisons were performed for the statistical analysis (∗*p* < 0.05; ∗∗*p* < 0.01; ∗∗∗*p* < 0.001). For ELISA results, One‐way ANOVA and Kruskal–Wallis multiple comparisons test were performed for the statistical analysis (∗*p* < 0.05; ∗∗*p* < 0.01 vs. TNF*α*‐treated samples; °*p* < 0.05; °°*p* < 0.01 vs. untreated samples). For oxidative stress evaluation the Student *t*‐test was performed for statistical significance ∗*p* < 0.05; ∗∗*p* < 0.01; ∗∗∗*p* < 0.001 vs. untreated samples; °*p* < 0.05; °°*p* < 0.01; °°°*p* < 0.001 vs. TNF*α*‐treated samples.

In NHDF, both treatments (MUD and TW) statistically and significantly (*p* < 0.001) reduced the gene expression of the cytokines IL1*β* and IL6. MUD was slightly more effective on IL6, whereas TW showed a greater reduction on IL1*β*. The expression of genes related to the inflammatory enzymes COX2 and iNOS showed a downward trend, but the change was not statistically significant.

The expression of HO1, an antioxidant and protective marker, increased significantly (*p* = 0.026) in the presence of the treatments, especially with TW (*p* < 0.05). This indicates the activation of a protective cellular response (Figure 4a).

Consistent with the genetic data, TNF*α* increased the levels of these cytokines, especially IL6, and the level of protein production. Treatment with MUD significantly reduced the secretion of both, returning them to levels similar to the control (CTRL). TW showed a lesser protective effect on IL6 than MUD (Figure 4b).

No significant changes were observed between control, TNF*α*, and treatments, suggesting that the stimulus or treatments did not directly influence overall ROS production at this specific time point (Figure 4c). TNF*α* dramatically reduced the activity of the protective enzyme SOD (*p* < 0.001), whereas treatments with MUD and 5% TW successfully restored SOD activity, with TW showing the greatest effect, restoring it to higher levels than CTRL (*p* < 0.001) (Figure 4c). GSH levels remained stable and did not show significant variations between the different conditions (Figure 4c). In fibroblast cells, it is evident that both MUD and TW possess anti‐inflammatory properties, as they reduced the expression and release of the key cytokines IL1*β* and IL6 induced by TNF*α*. Furthermore, although the treatments do not reduce total ROS or GSH, they act by enhancing the cell′s antioxidant defenses, stimulating the expression of HO1 and restoring the activity of the SOD enzyme.

For the keratinocyte line (Figure 5), the effect is differentiated. For IL1*β*, only TW shows a significant reduction (*p* < 0.01). For IL6, MUD has no effect, whereas TW drastically reduces gene expression (*p* < 0.001). COX2 also shows a statistically significant variation in the presence of MUD, which significantly reduces COX2 (*p* < 0.05), whereas TW does not show a relevant effect. Global changes for iNOS and HO1 are not statistically significant. However, there is a tendency for MUD to reduce them, which is not observed with TW (Figure 5a). TNF*α* significantly increases the secretion of both IL1*β* and IL6 cytokines (*p* < 0.01). In this case, both MUD and TW significantly and progressively reduce the protein levels of IL1*β* and IL6, although not completely returning them to basal control levels (CTRL) (Figure 5b). Regarding the evaluation of oxidative stress on HaCaT, TNF*α* doubles ROS levels compared with CTRL (*p* < 0.001), and both treatments (MUD 5% and TW 5%) are extremely effective in reducing oxidative stress (*p* < 0.01), significantly lowering ROS production. Moreover, TNF*α* halves SOD activity (*p* < 0.001) and the treatments (MUD 5% and TW 5%) completely restore the enzymatic activity bringing it to control levels (with MUD showing a slight increase, *p* < 0.001). GSH levels remain essentially stable, without significant variations between groups (Figure 5c). Both treatments show notable activity at the protein level, reducing inflammation (IL1*β* and IL6) and effectively counteracting oxidative stress induced by TNF*α*.

### 3.5. Effects of MUD and TW Extracts on Cell Migration and Gene Expression

Cell migration is an essential component of the wound‐healing process. To investigate whether MUD and TW extracts could influence wound closure under inflammatory conditions, an in vitro scratch assay was performed in TNF‐*α*–pretreated NHDF and HaCaT cells. Following mechanical injury, cells were treated with 5% MUD or 5% TW, and wound closure was monitored at 6 and 24 h.

In NHDF cells, both MUD and TW significantly promoted wound closure compared with the TNF‐*α*–treated control at 6 h (*p* = 0.002). The reduction in wound area was already evident at this early time point, indicating that both treatments enhanced the cellular response to the scratch injury under inflammatory conditions (Figure 6a,b).

Figure 6Effects of MUD and TW extracts on in vitro wound closure and inflammatory gene expression. (a–b) NHDF cells: Cells were seeded in sixwell plates and a linear scratch was performed with a 10‐*μ*L tip. (a) Representative images of the wound area at 0, 6, and 24 h after wounding and treatment with 5% MUD or TW. The scale bar is the same for all images. (b) Quantification of wound repair expressed as percentage of closure relative to T0 (100% free area). Data represent mean ± SD of two representative experiments. Statistical significance was determined by Student *t*‐test: ∗∗∗*p* < 0.001 vs. T6; °°°*p* < 0.001 vs. T0; §§ TNF‐*α* + MUD vs. TNF‐*α* *p* = 0.002; ## TNF‐*α* + TW vs. TNF‐*α* *p* = 0.002. (c–d) HaCaT cells: Cells were treated and analyzed as described for NHDF. (c) Representative images of wound area at 0, 6, and 24 h after wounding and treatment. (d) Quantification of wound closure percentage relative to T0. Statistical significance: ∗∗∗*p* < 0.001 vs. T6; °°°*p* < 0.001 vs. T0; § TNF‐*α* + MUD vs. TNF‐*α* *p* = 0.017; # TNF‐*α* + TW vs. TNF‐*α* *p* = 0.027. (e–f) Inflammatory gene expression by qPCR in scratched, TNF‐*α*–stimulated cells treated with 5% MUD or TW. (e) NHDF: IL‐1*β*, IL‐6, COX‐2, iNOS, and HO‐1 mRNA levels. (f) HaCaT: IL‐1*β*, COX‐2, iNOS, and HO‐1 mRNA levels. Data are expressed as mean ± SEM of two independent experiments in duplicates. One‐way ANOVA followed by Kruskal–Wallis multiple comparisons test was used for statistical analysis: ∗*p* < 0.05; ∗∗*p* < 0.01; ∗∗∗*p* < 0.001.

**Figure:**
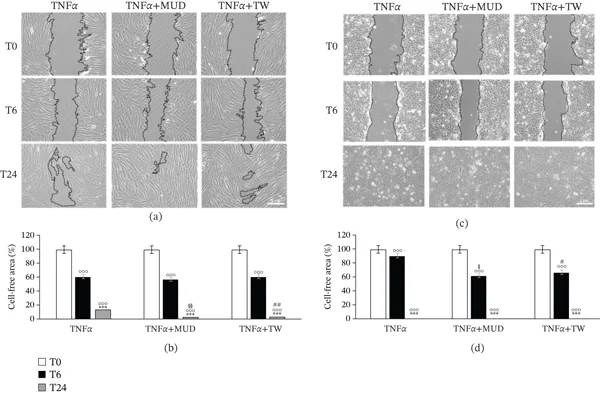

**Figure:**
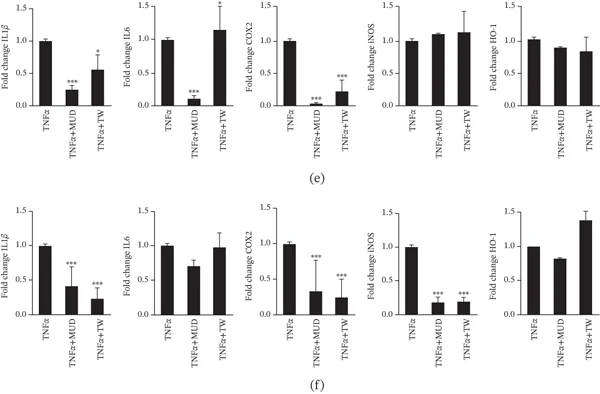

In HaCaT cells, treatment with MUD and TW similarly resulted in a significant reduction in wound area at 6 h compared with TNF‐*α*–treated cells (*p* = 0.017 and *p* = 0.027, respectively). Thus, both preparations accelerated the early phase of wound closure in the two skin cell models, although the magnitude of the response differs between cell types (Figure 6c,d).

At 24 h, complete wound closure was observed under all experimental conditions, indicating that the differences between treatments were mainly evident during the early phase of the repair process. These findings suggest that MUD and TW may facilitate early cellular migration and wound closure in vitro, particularly under TNF‐*α*–induced inflammatory conditions. However, because complete closure was observed at 24 h in all groups, the present results support an effect on the kinetics of wound closure rather than demonstrating a sustained enhancement of the overall healing capacity.

To investigate the mechanisms underlying the prohealing effects of MUD and TW extracts, we analyzed their impact on inflammatory gene expressions in scratched, TNF‐*α*–stimulated NHDF and HaCaT monolayers. After 6 h of TNF*α* pretreatment and mechanical injury, cells were incubated with MUD or TW until 24 h, and the mRNA expression levels of IL‐1*β*, IL‐6, COX‐2, iNOS, and HO‐1 were evaluated by qPCR.

In NHDF cells, MUD treatment significantly reduced the expression of IL‐1*β*, IL‐6, and COX‐2 (*p* < 0.001), whereas no significant changes were observed in iNOS and HO‐1 levels. TW treatment also significantly downregulated IL‐1*β* (*p* < 0.05), IL‐6 (*p* < 0.05), and COX‐2 (*p* < 0.001), and induced a slight, although not statistically significant, increase in iNOS expression (Figure 6e).

Similarly, in HaCaT cells, both MUD and TW significantly reduced the expression of IL‐1*β*, COX‐2, and iNOS (*p* < 0.001). Moreover, TW treatment significantly increased HO‐1 expression in TNF*α*‐stimulated HaCaT cells (*p* < 0.05), suggesting a combined anti‐inflammatory and antioxidant effect during the wound healing process (Figure 6f).

## 4. Discussion

Saturnia spa water and mud aqueous extract are complementary approaches to conventional drug therapies, offering benefits in a range of skin conditions. They have been shown to alleviate symptoms of several chronic inflammatory conditions, including psoriasis, atopic dermatitis [21, 22], acne vulgaris [23], and wound healing [24, 25].

The effects and efficacy of spa treatments are attributed to the combined antioxidant, anti‐inflammatory, and analgesic properties derived from their unique physical and biochemical characteristics. These include the regulation of cytokine release, NF‐*κ*B expression and activity, and adhesion molecule expression [26, 27].

This study evaluated the anti‐inflammatory properties of MUD and TW from Saturnia Spa in vitro models of skin inflammation. Keratinocytes (HaCaT) and fibroblasts (NHDF) pretreated with TNF*α* represent a well‐established model for assessing the potential therapeutic effects of TW or MUD in inflammatory skin diseases [28, 29].

From a chemical perspective, Saturnia mud exhibits a siliceous–aluminous composition enriched with trace elements, whereas the associated Saturnia spa water is highly mineralized and classified as a calcium–sodium sulfate water. It contains elevated concentrations of calcium (605.6 mg L^−1^), sodium (94.77 mg L^−1^), magnesium (59.75 mg L^−1^), and sulfur (> 650 mg L^−1^), consistent with deep‐circulating hydrothermal systems and in agreement with established hydrogeochemical models of European thermal springs [18, 19]. The trace element profile, including lithium, strontium, fluoride, boron, and silicon (12.89 mg L^−1^), reflects prolonged interaction with carbonate and silicate lithologies, whereas the absence of detectable iron suggests reducing geochemical conditions [30].

The combination of clay minerals, crystalline phases, and dissolved ions likely contributes to the observed biological effects. In particular, the presence of sulfur species and trace elements, together with the high cation content, may support redox balance and modulate inflammatory responses, as reported for other mineral‐rich peloids and thermal waters [31–33].

Following treatment with MUD and TW, TNF*α*‐activated keratinocytes and fibroblasts exhibited downregulation of IL1*β*, IL6, and COX2 at both the gene and protein levels, consistent with findings reported by Dal Pra et al. and other studies [34–36]. These results indicate an attenuation of the proinflammatory cytokine cascade typically triggered by TNF*α* stimulation in these cells. This serves as a proof of concept for the direct anti‐inflammatory and immunomodulatory effects of thermal derivatives in skin disease contexts. Both mud aqueous extract and Saturnia spa water attenuated TNF‐*α*–induced inflammatory responses in keratinocytes and fibroblasts. The pronounced suppression of IL‐1*β*, IL‐6, COX‐2, and iNOS by MUD suggests greater relevance to psoriasis‐like inflammation, whereas the ability of TW to induce HO‐1 in keratinocytes indicates additional antioxidant and cytoprotective effects, potentially beneficial in chronic skin conditions and wound repair.

Additionally, mud aqueous extract and Saturnia spa water demonstrated efficacy in counteracting oxidative stress in keratinocytes and fibroblasts. Skin exposure to endogenous or exogenous stimuli often results in oxidative stress and ROS overproduction, compromising antioxidant defenses. An inability to neutralize ROS may alter gene expression linked to inflammation, immune response, cell cycle, and viability. Chronic oxidative stress and persistent skin inflammation are key contributors to skin damage, premature aging (inflammaging), increased cancer risk, and the chronicity of inflammatory skin disorders. To mitigate ROS‐induced damage, antioxidant molecules like HO‐1, GSH, and SOD are essential [37–39]. Our results clearly demonstrate the antioxidant effects of MUD and TW against ROS, as evidenced by the upregulation of SOD and GSH activities, thereby confirming their antioxidant potential.

The anti‐inflammatory and cytoprotective effects of the HO system have been observed in HaCaT and NHDF cells, with increased HO‐1 expression shown to confer protection in disease models [40–42]. The greater responsiveness of HaCaT cells observed in our study is consistent with previous reports indicating high HO‐1 expression in keratinocytes, whereas fibroblasts require external stimuli to induce lower levels of expression [43].

Considering the modulatory role of MUD and TW on HO‐1, we also assessed GSH and SOD levels in TNF‐*α*–treated NHDF and HaCaT cells. Our findings showed that MUD and TW treatment enhanced SOD levels, indicating their capacity to modulate the cellular redox balance.

Altogether, our results suggest that Saturnia TW, by increasing HO‐1 and SOD levels and reducing proinflammatory cytokine release in TNF‐*α*–treated skin cells, may improve cellular function by mitigating oxidative stress, inhibiting excessive inflammation, and restoring physiological homeostasis. Inducible HO‐1 is known to regulate multiple processes relevant to wound healing and protection against apoptosis [44–46].

One limitation of this study is that all experiments were performed solely under in vitro conditions. Further clinical research on specific skin conditions is needed to correlate these findings with potential therapeutic outcomes.

In conclusion, our data suggest that derivatives from Saturnia Spa could offer benefits for skin disorders characterized by oxidative stress and chronic inflammation. These products may serve as coadjuvants to conventional treatments or as cosmeceutical supports, due to their ability to limit inflammation and restore redox balance, thereby reestablishing skin homeostasis.

## 5. Conclusion

In conclusion, our results demonstrate that MUD and TW exhibit anti‐inflammatory and redox‐modulating effects in TNF‐*α*–stimulated human keratinocytes and dermal fibroblasts. Both treatments reduced the expression and/or secretion of inflammatory cytokines, while modulating cellular antioxidant responses through HO‐1 and SOD activity. In HaCaT cells, MUD and TW also significantly reduced TNF‐*α*–induced ROS levels, whereas GSH levels remained unchanged

Importantly, both treatments promoted early wound closure in the in vitro scratch assay, suggesting a potential role in modulating cellular responses involved in skin repair. Overall, these findings highlight the biological activity of Saturnia water and mud extract under the experimental conditions investigated and provide a basis for further studies to clarify the mechanisms underlying their effects and their potential applications in skin repair.

## Author Contributions

E.C. and B.S. designed the experimental study. E.C., C.S., and S.K. performed experiments. G.T. and M.S. analyzed the data. E.C., G.P., M.R., and B.S. wrote the article. B.S. supervised this study.

## Funding

No funding was received for this manuscript. Open access publishing facilitated by Universita degli Studi Gabriele d’Annunzio Chieti Pescara, as part of the Wiley ‐ CRUI‐CARE agreement.

## Disclosure

All authors have reviewed and approved the final version of the manuscript.

## Ethics Statement

The authors have nothing to report.

## Consent

The authors have nothing to report.

## Conflicts of Interest

The authors declare no conflicts of interest.

## Data Availability

The data that support the findings of this study are available from the corresponding author upon reasonable request.
